# Synaptic Interactions in Scorpion Peg Sensilla Appear to Maintain Chemosensory Neurons within Dynamic Firing Range

**DOI:** 10.3390/insects12100904

**Published:** 2021-10-03

**Authors:** Douglas D. Gaffin, Safra F. Shakir

**Affiliations:** Department of Biology, University of Oklahoma, Norman, OK 73019, USA; Safra.F.Shakir-1@ou.edu

**Keywords:** pectines, navigation, electrophysiology

## Abstract

**Simple Summary:**

Scorpions have unusual taste organs called pectines that they drag over the ground as they walk. Minute, peg-shaped sensilla adorn the ground-facing surfaces of the pectines, and each of these “pegs” contains several chemosensitive neurons and at least one mechanosensitive neuron. Of particular interest is that some of these neurons interact synaptically at the level of the peg sensillum prior to relay to the scorpion brain. Here we use a technique called “conditional cross-interval correlation analysis” to show that heightened activity of two of the neurons appears to induce a third neuron, which in turn inhibits the previous two. We suggest that the dynamics of this simple feedback circuit might serve to maintain the sensory neurons in a sensitive range so that substrate information can be accurately detected and processed, such as during tracking sexual pheromone trails and/or recapitulating home-directed training paths.

**Abstract:**

Scorpions have elaborate chemo-tactile organs called pectines on their ventral mesosoma. The teeth of the comb-like pectines support thousands of minute projections called peg sensilla (a.k.a. “pegs”), each containing approximately 10 chemosensory neurons. Males use pectines to detect pheromones released by females, and both sexes apparently use pectines to find prey and navigate to home retreats. Electrophysiological recordings from pegs of *Paruroctonus utahensis* reveal three spontaneously active cells (A_1_, A_2_, and B), which appear to interact synaptically. We made long-term extracellular recordings from the bases of peg sensilla and used a combination of conditional cross-interval and conditional interspike-interval analyses to assess the temporal dynamics of the A and B spike trains. Like previous studies, we found that A cells are inhibited by B cells for tens of milliseconds. However, after normalizing our records, we also found clear evidence that the A cells excite the B cells. This simple local circuit appears to maintain the A cells in a dynamic firing range and may have important implications for tracking pheromonal trails and sensing substrate chemistry for navigation.

## 1. Introduction

Pectines are complex chemo-tactile organs located on the ventral side of the scorpion mesosoma. The pectines are composed of teeth that each contain tens to hundreds of microscopic peg sensilla that detect stimuli when they brush against the ground [1,2]. Note: In this article we follow nomenclature used by Foelix and Müller-Vorholt (1983) where the “pectines” are the paired, comb-like appendages that hang from the scorpion’s ventral mesosoma, the “teeth” are the stout projections that extend from the pectinal spine (similar to teeth or tines of a comb), and the “pegs” are the tens to hundreds of peg sensilla that adorn the distal, ground facing surfaces of each tooth. The peg sensilla contain both chemosensory and mechanosensory receptors [3,4]. It appears that most of the pegs along a pecten respond identically to the same chemicals [5]. Pectines seem to help the scorpions identify chemical stimuli for tracking both prey [6] and mates [7,8]. Questions have been raised as to what other functions the pectines could serve. Nightly hunting excursions take scorpions far away from their burrows, but they still find a way to return to them. The *Navigation by Chemotextural Familiarity Hypothesis* (NCFH) suggests that scorpions learn chemical and textural information gathered when the pectines brush against the ground that is used during subsequent returns home [9,10].

Previous electrophysiological research on the peg sensilla of the sand scorpions *Paruroctonus utahensis* and *Smeringurus mesaensis* revealed that the pegs contain three spontaneously active cells, named A_1_, A_2_, and B [1,5,11,12]. The peg sensilla contain additional neurons, including a mechanosensory neuron that fires when the peg is deflected. The mechanosensory waveforms are triphasic, quickly adapting, and decidedly larger in amplitude than the A and B waveforms [1]. Although morphological accounts show there are additional (at least 10) neurons resembling chemosensitive cells in the pegs, additional waveforms have appeared only occasionally in electrophysiological studies [1,2]. In all previous recordings, the A and B waveforms are spontaneously active and responsive to chemical stimulation. The A_1_ and A_2_ waveforms are triphasic and distinguishable only by amplitude, which is dependent on electrode position relative to the spike generation zone. Often the two waveforms superimpose and are not digitally resolvable. In recordings where the two waveforms resolve, the A_1_ label is given to the larger amplitude spike. The B cell is readily resolvable by its small amplitude, biphasic waveform [1,11].

Of particular interest, the A_1_, A_2_, and B cells appear to interact synaptically, which is noteworthy because peripheral synaptic interactions seem relatively rare in arthropod chemoreceptors, though they are more represented among the chelicerates compared to the mandibulates [13,14,15,16,17]. Most notably, the activity of the B cells inhibits the A cells for tens of milliseconds, followed by a post-inhibitory rebound of A cell activity at about 100 ms. It has been suggested that this local network may serve to enhance sensory information [2], similar to lateral inhibitory circuits in various sensory systems [18,19,20]. However, during long baseline recordings, or with increased A cell activity through stimulation, cross-correlograms of A cells relative to the B cell show not only an inhibitory effect of B on A, but also a peak in the A cells just prior to the firing of the B cell [2]. This suggests that there might be an excitatory effect of the A cells onto the B cell.

This latter observation raises additional questions about the function of these synaptic interactions in peg sensilla. It seems possible that the A cells could be the main carriers of the chemosensory information and that the B cells serve as a governor to maintain the A cells within a sensitive range, such as during tracking of pheromonal signals by males [7,8,21]. However, both male and female scorpions have pectines, and this synaptic interaction is present in peg sensilla of both sexes [2]. The NCFH suggests that the matrices of peg sensilla on pectines are important for acquiring accurate chemical and textural information about their habitat [9,10]. There is also evidence that scorpions use their pectines to track prey [6,22]. The governor idea would seem useful in either navigation or hunting in helping avoid sensory adaptation.

No matter what the larger context may be—information enhancement, mate tracking, homing, or other—here we systematically investigate the excitatory effect of A cells on B cells. We made long-term, extracellular recordings of spontaneous activity of peg sensilla and carefully isolated the three cell types—A_1_, A_2_, and B. We then applied conditional cross-interval correlation analyses [23] to assess the near-term interactions of A and B cells. We found that after normalizing by background spiking frequency, the activity of the A cells is elevated immediately preceding the firing of the B cell. These findings suggest the existence of a governor relationship between the A and B cells in which increased activity of the A cells calls up the B cells, which in turn suppress the activity of the A cells.

## 2. Materials and Methods

### 2.1. Collection of Scorpions

Desert grassland scorpions (*P. utahensis*) were captured at night from sandy areas near Monahans, Texas. The scorpions were stored in glass jars that contained sufficient sand for burrowing and a shard of pottery to use for shelter. They were fed one cricket every two weeks and were given water twice weekly. The scorpions’ light cycle was reversed to ensure that the electrophysiological recordings of neural activity occurred during the most active part of their circadian cycle. The animals were adapted to the reversed light regime for several weeks prior to recording. In all, we recorded from seven female (*P. utahensis*) for a total of 25.8 h. We recorded from multiple pegs for several of the animals and from both right and left pectines. We used only females to control for any potential differences between the sexes. For our detailed analyses, we picked recordings based on their longevity, stability, and exceptional signal-to-noise ratio. After recording, each scorpion was returned to its jar if it survived or was euthanized in the freezer if there was significant hemolymph lost or if the animal was not in good health.

### 2.2. Preparation for Recording

**Figure 1:**
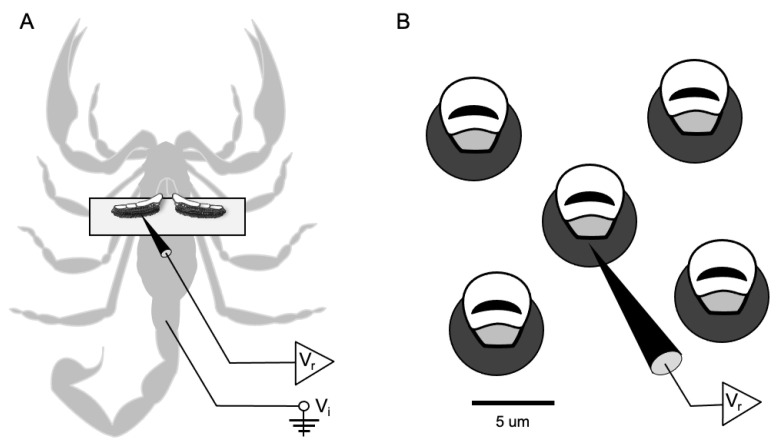
Extracellular electrophysiological recording from individual peg sensilla. (**A**) The scorpion is shown ventral side up with the pectines secured atop a coverslip. Note the recording electrode (V_r_) inserted into the left pecten and the indifferent electrode (V_i_) inserted in the tail. (**B**) A close up diagram of individual peg sensilla shows the recording electrode inserted through the base of a single peg.

Each scorpion was recorded using the same method. The scorpion was removed from its jar and placed in the freezer for approximately 1–2 min. Once immobilized, the animal was placed ventral side up on a microscope slide to allow access to the pectines. The stinger, legs, and pedipalps were all restrained using modeling clay. The animal was placed under a microscope at 40*x* power, and the pectines were adhered to half of a coverslip with double-sided tape (Figure 1). This coverslip was held in position with the clay. The end of a length of silver wire was sharpened and inserted into the tail to access the hemolymph and serve as the indifferent electrode (V_i_). The wire was fastened in the clay to maintain a strong connection with the hemolymph while allowing ample access to the free end for subsequent connection to the amplifier. Recording electrodes (V_r_) were electrolytically carved from tungsten by passing current through the wire inserted in a 1M sodium nitrate solution and connected to a carbon anode. The electrodes were shaped to a tip diameter of about 1µm, narrow enough to insert into the base of individual peg sensilla.

The prepared scorpion was moved and fastened to a stage under a compound microscope inside a Faraday cage. The table for the cage floated on nitrogen gas to minimize vibrations and electrical noise. We viewed the peg sensilla using an Olympus BX-50WI microscope (up to 1250*x*) equipped with epi-illumination and long working distance objectives; we found a magnification of 500–1000*x* to be the most effective for visibility. The tungsten recording electrode was fastened to a Leitz micromanipulator and inserted into the base of individual peg sensilla (Figure 1). The recording electrode was connected to a differential amplifier (DAM 80, World Precision Instruments, Sarasota, FL, USA) that boosted the signal by 1000*x* to 10,000*x*; the signal was bandpass filtered between 300 Hz and 1 kHz and visualized on an oscilloscope (Techtronix Dual Beam Storage Oscilloscope). The action potentials were also audiblized with an amplifier (Pule PTA2 Stereo Power Amplifier) and speakers (Realistic 7W). Finally, the signal was digitized (1401-micro 3, Cambridge Electronics Design, Cambridge, UK) at a sampling rate of 15 kHz to create the raw records.

### 2.3. Processing of Action Potentials

We feel confident that the signals we recorded emanated from neurons within the impaled sensillum. Pore-tip recordings using electrolyte-filled glass electrodes of pectines submerged in mineral oil show the same types of waveforms we saw in our recordings [5,24,25], which further corroborates that we were accessing neurons resident to the sensillum and not from neighboring sensilla. We saved the raw records using Spike2 software (Cambridge Electronics Design) and isolated the action potentials from background noise using the “New Wavemark” module. The data were then transferred from Spike2 to a specially created MATLAB program (Figure S1) to accurately classify the A_1_, A_2_, and B spikes. In MATLAB we parsed the spikes sampled at 15 kHz to 45 points (3 ms width), the fewest number of points necessary to accurately encapsulate the full shape of the waveforms (see expanded waves in Figure S1). To clean the records, we analyzed 100 s at a time by expanding each segment and displaying the superimposed waveforms in an expanded time window. We used the cursor to tag a portion of the B spike waveforms in a region clear of other spike types. This process initially sorted the record into two categories: B spikes (shown as red in Figure S1) and all other spike types (shown as blue). This process was iterated to the end of the record to generate the first pass sort. This entire process was then repeated separately on the two groups of spikes to generate the second pass sort, which yielded pure A_1_, A_2_, and B spikes. The accuracy of the groupings was analyzed via auto-correlation analysis (described below).

### 2.4. Auto- and Cross-Correlation Analysis

Auto-correlation analysis was used to confirm the classification of the A_1_, A_2_, and B spikes [26], essentially determining if the classified waveforms are from one cell or from more than one cell. We graphed the occurrence of all of one set of waveforms against themselves in small windows of time. The time window of the histogram is represented in the x-axis, and the number of spikes in the y-axis; the referenced spikes are centered at time zero. If a spike category actually contains only one spike type, there should be no waveforms near the referenced spikes (time zero) owing to the refractory period. If the analysis shows waveforms around the zero point, there is another cell type in the set of waveforms and the classification is inaccurate. The auto-correlation analysis confirmed the A_1_, A_2_, and B spike classifications yielded by the final sorts.

We used cross-correlation analysis [27,28] to determine if the A and B cells were interacting. In this analysis, spikes from two cells are graphed against each other in small windows of time. Superimposing all of the windows in the record generates a histogram. In the example shown in (Figure S2), the occurrence of spikes of type A_2_ have been graphed relative to spikes of type B and the activity of the A_2_ spikes are diminished for several milliseconds after the occurrence of the B spikes, suggesting that the B spikes have an inhibitory effect on the A_2_ spikes.

### 2.5. Conditional Interspike Interval and Conditional Cross-Interval Plots

Following the final sorts, we wrote another MATLAB script to display the data in two plots: a Conditional Interspike Interval (CII) plot and a Conditional Cross-Interval (CCI) plot [23]. The description for how these two types of plots are created is described in Figure S3.

## 3. Results

**Figure 2:**
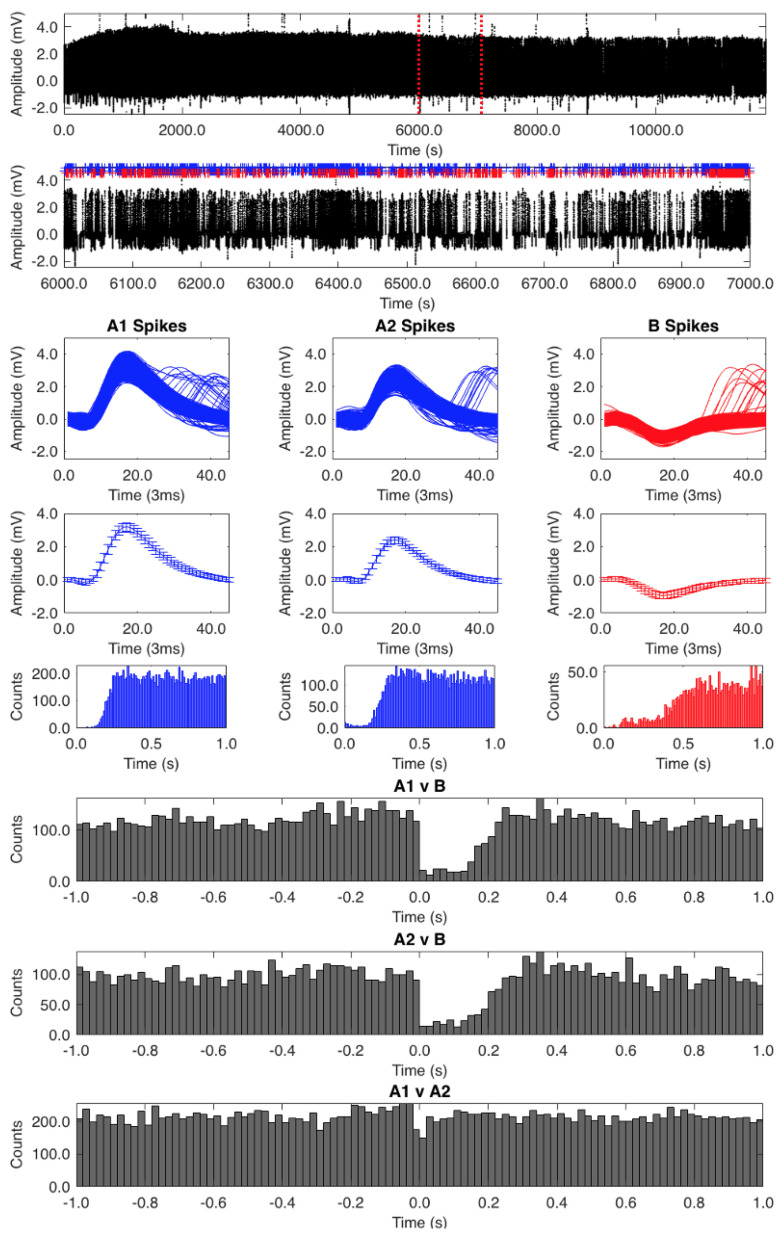
Auto- and cross-correlation analysis. The top trace shows the unaltered record containing all unmarked spikes. The second trace shows an expanded 1000 s segment between the red dotted lines in the top trace. The uppermost square windows show the superimposed A_1_, A_2_, and B spike waveforms, below which are the average spike waveforms (+/− SD). Below these are the auto-correlograms for each spike classification. The bottom three histograms are cross-correlograms for the A_1_ vs. B, A_2_ vs. B, and A_1_ vs. A_2_ spikes.

The auto-correlation analysis of one of our records (low spiking frequency of 2 Hz) showed that we had isolated appropriate classifications of the A_1_, A_2_, and B spikes (Figure 2). The A_1_ spike auto-correlation analysis was the most accurate, with no waveforms at the zero point. The A_2_ auto-correlation analysis showed a few waveforms around the zero point, which could be attributed to some A_1_ spikes being misclassified. The B spikes had a few waveforms around the zero point too but largely represented a pure classification.

Cross-correlograms (Figure 2, bottom three histograms) of A_1_ vs. B and A_2_ vs. B show a clear inhibitory effect of the B cell on the A cells for the first 0.2 s following B cell activity. Cross-correlograms of A_1_ vs. A_2_ show no evidence of mutual influence. Though not overtly obvious, there does appear to be a slight ramping up of A cell activity (most noticeable in the A_1_ vs. B correlogram) prior to B cell activity.

**Figure 3:**
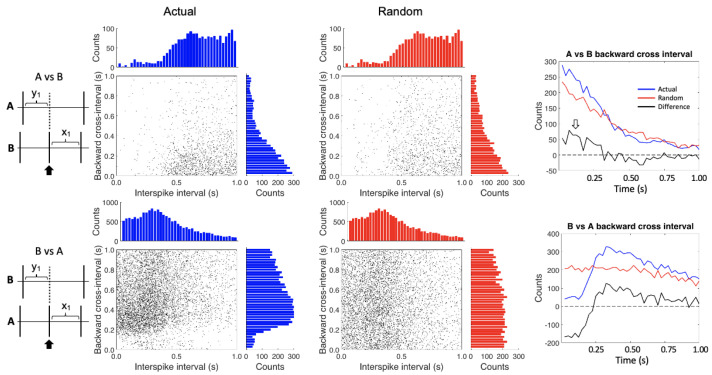
Conditional interspike interval plots. In the upper plots, the A spikes are referenced against the B spikes while the reverse is shown in the lower plots. The left hand (blue) plots show the actual data while the right hand (red) plots were created after randomizing the interspike intervals for each of the spike trains. The histograms on the tops and the sides of the plots show the counts by time bin for each axis–essentially compressing the x-y axes on their respective axis. The two graphs on the right show the differences (black lines) between the actual (blue lines) and randomized data (red lines) for the backward cross intervals. The open white arrow highlights the area of increased activity in the difference plot of the A vs. B backward cross-interval graph.

Conditional spike interval analysis assesses the near-temporal interactions between spike trains. The conditional interspike-interval (CII) plots are shown in Figure 3. For clarity, we have combined the A_1_ and A_2_ cells, but each shows the same patterns individually. In the top plots the A spikes are the cross train and the B spikes the reference train. The histograms on the tops of the plots show the interspike intervals by time bin. As expected, the refractory period for the B spikes is recovered in the upper histograms of the A vs. B plots. The histograms on the sides show the various backward cross intervals by time bins. Note the ramping up of A cell activity right before the B spike firing in the A vs. B “Actual” plot.

In the bottom plots, the B spikes are the cross train and the A spikes the reference train. Note that in the top two histograms, the refractory period does not fall to zero because we have combined the two A spikes (they do fall to zero when the A_1_ and A_2_ spikes are analyzed separately, not shown). The backward cross interval histogram on the right side of the B vs. A “Actual” plot shows a lack of B spikes just before the A cell firing, which reflects the inhibitory effect of the B cell on the A cells.

It is important to control for any inherent spiking patterns in the spike trains, so we randomized the interspike intervals for each of the spike trains and reapplied the analysis (Figure 3, “Random” plots). The top histograms are the same as for the non-randomized data because the distribution of interspike intervals does not change, even after randomization. Note that the A spike backward cross interval activity is diminished compared to the non-randomized data in the A vs. B plot.

The two summary graphs on the right side of Figure 3 show the difference between the actual and randomized data for the backward cross intervals (plotted as a black line). After normalizing, the A vs. B backward cross interval plot shows an increase of the A spikes in the first 0.25 s before B spike firing (white arrow). The B vs. A backward cross interval plot highlights the inhibitory effect of the B cell on the A cells, along with a post inhibitory rebound.

**Figure 4:**
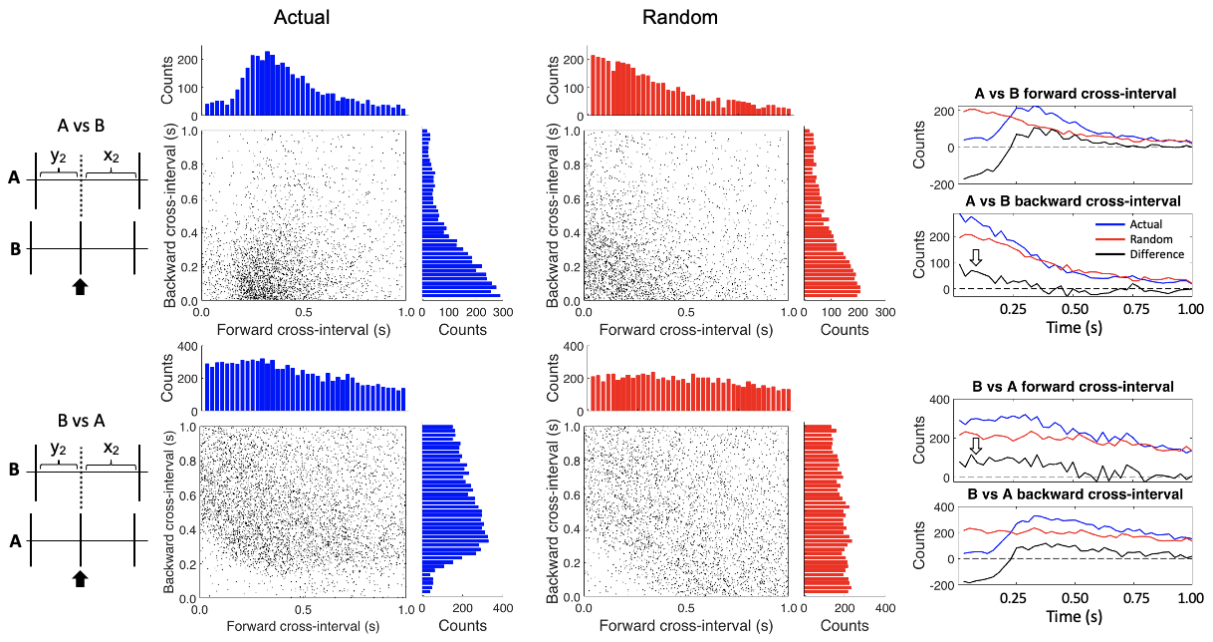
Conditional cross-interval plots. The A spikes are referenced against the B spikes in the upper plots while the reverse is true in the lower plots. The left hand (blue) plots show the actual data while the right hand (red) plots were created after randomizing the interspike intervals for each of the spike trains. The histograms on the tops and the sides of the plots show the counts by time bin for each axis. The four graphs on the right show the differences (black lines) between the actual (blue lines) and randomized data (red lines) for both the forward and backward cross intervals. The open white arrows highlight areas of increased activity in the difference plots.

The conditional cross-interval analysis further confirmed these relationships (Figure 4). In the upper histograms the “Actual” and “Random” graphs differ because instead of randomizing the same train intervals (as we did in CII) we are randomizing the intervals of the opposite, cross train; as such, all the relationships change. Therefore four summary graphs (at right) are required to assess these relationships. First, after subtracting the randomized data, we again see the inhibition and post-inhibitory rebound effect of the B cells on the A cells in the A vs. B forward cross-interval and the B vs. A backward cross-interval graphs. Furthermore, the apparent excitatory effect of the A cells on the B cells (white arrows) is seen in the A vs. B backward cross-interval graph (heightened occurrence of A cells just before B cell firing) and B vs. A forward cross-interval graph (heightened occurrence of B cells just after A cell firing).

We also applied auto- and cross-correlation analysis on other records, including a higher frequency baseline recording (19 Hz). The A spikes did not resolve in this recording and are combined in the results shown in Figure S4. The cross-correlogram of the A spikes vs. B spikes shows a peak of activity of the A spikes immediately prior to the firing of the B cells (* in Figure S4). The CII analysis of this record (Figure S5) shows a pronounced peak in the difference between the actual and randomized data of the A vs. B backward cross interval in the 0.25 s prior to spike B activity (white arrow).

## 4. Discussion

**Figure 5:**
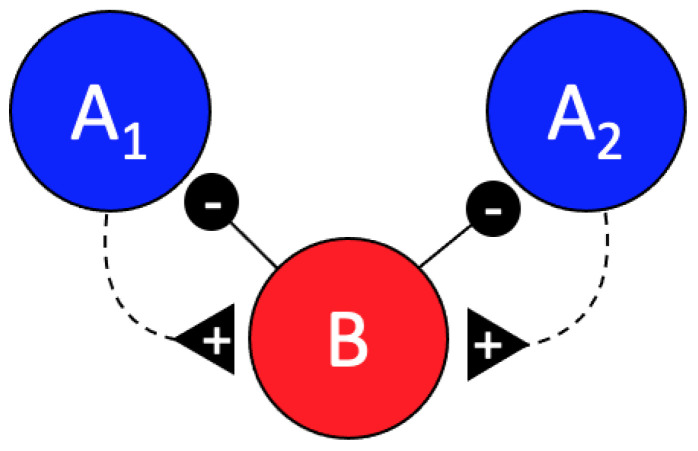
Putative feedback circuit configuration. Increased activity of the A_1_ and A_2_ cells appears to directly or indirectly induce activity of the B cell, which in turn inhibits the firing of the A cells.

In addition to the previously described inhibitory effect of the B cell on the A cells in scorpion peg sensilla, we found strong evidence of an excitatory effect of the A cells on the B cell. In particular, our findings in the conditional interspike-interval and conditional cross-interval analyses showed that activity of the B cells is increased during the first quarter second following the activity of the A cells. This is most notable in the forward cross interval plots of spike B in relation to the A spikes after normalizing by subtracting the randomized background interspike intervals (Figure 3, B vs. A forward cross interval). These data suggest a simple feedback circuit as shown in Figure 5.

**Figure 6:**
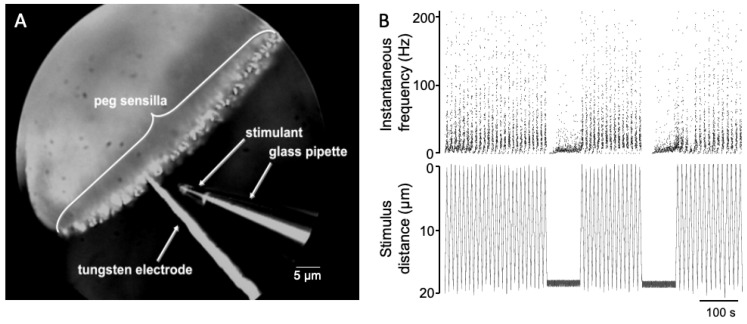
Spiking activity closely follows travel of stimulus pipette. (**A**) Example of a repeated stimulation with a hexanol-filled pipette moved between 0 and 20 microns of the tip of a recorded peg sensillum. (**B**) Instantaneous spiking frequency of combined peg sensillum neurons (top) and distance of pipette from peg tip (bottom).

What could be the significance of such a local circuit? The pectines are an important sensory organ for scorpions. Peg sensilla number in the tens of thousands in some species, with each peg containing 10 or more energetically expensive sensory neurons [3,4]. In fact, the neural projection from peg sensilla is extensive, topographically arranged, and represents the largest input to the scorpion central nervous system [29,30,31,32]. Previous recordings from peg sensilla using a technique of recording from pectines submerged in mineral oil using electrolyte filled glass pipettes indicate that the neural responses of the A cells are largely similar among the peg population [5,24,25,33]. It seems possible that a simple, local feedback circuit helps prevent sensory adaptation [34] of the A cells. In this scenario, increased activity of the A cells activates the B cells, which in turn suppress the A cell activity, perhaps keeping them within a dynamic sensory range somewhat similar to that seen in bee taste neurons [35]. In line with this reasoning, it is interesting that the combined frequencies of the A_1_ and A_2_ spikes do not exceed 90 Hz, even under constant, direct chemical stimulation of the peg tip [5]. Studies involving stimulation of peg sensilla with a pure chemical stimulant that was repeatedly moved near to a recorded peg (see Figure 6 and [36]) showed that the activity of the A spikes tracked the stimulant distance very well (the different distances would represent different chemical concentrations). It seems possible that the accuracy of spiking frequency in relation to chemical concentration may be a product of these synaptic interactions.

In previous studies, we suggested that peg inhibitory synapses may be important for enhanced detection and processing of chemical stimuli [2]. While still possible, such information enhancement circuits are often arranged to increase spatial resolution and/or facilitate segmentation of the stimulus, such as in edge detection in visual and tactile systems [37,38,39]. To date, we do not know of physiological evidence of synaptic connections among sensory cells from neighboring peg sensilla—that is not to say that there might not be connections either in the pectinal tooth or at a higher level. Previous work shows clear axo-axonic synapses proximal to the sensory cell body layer about 100 microns below the tooth surface [3]. Additional morphological work hints at the potential for inter-connectivity among neurons from different sensilla [40]. As such, some form of contrast enhancement from adjacent sensilla is possible and needs to be examined.

However, as a result of the work presented here, we are thinking that instead of (or in addition to) sensory enhancement, the feedback circuit might help maintain the peg sensory neurons in a dynamic firing range. This could be important for accurate chemical tracking of prey or of pheromonal deposits without being overwhelmed by stimuli [7,8,21]. It could also be important for accurately learning the chemical make-up of the substrate around the scorpion’s home burrow for navigational purposes. The implications are not limited to scorpions. Interest in how various wandering arachnids home to their retreats is increasing [41,42,43,44,45,46,47], and it is notable that peripheral synaptic interactions seem prevalent in these animals [15,17,48,49].

The *Navigation by Chemotextural Familiarity Hypothesis* [9,10,42] predicts that scorpions use path integration or learning walks to acquire home-directed chemo-tactile information using the matrices of peg sensilla on their pectines. Then, during subsequent journeys they recapitulate these paths by comparing current pectinal “tastes” and “textures” with those most “familiar” in memory and moving in that direction. For this system to work, the neural responses of each peg sensillum must be accurate and consistent enough to allow for meaningful matrix comparisons. The feedback circuit described here may be important for this accuracy.

## Supplementary Materials

The following are available online at https://www.mdpi.com/article/10.3390/insects12100904/s1, Figure S1: Diagram of spike tagging process in MATLAB, Figure S2: Diagram of cross-correlation analysis, Figure S3: Creation of conditional interspike interval and conditional cross-interval plots. Figure S4: Auto and cross-correlation analysis of a higher spiking frequency record, Figure S5: Conditional interspike interval plots for higher frequency record.
