# Supplementary material for: Synaptic Interactions in Scorpion Peg Sensilla Appear to Maintain Chemosensory Neurons within Dynamic Firing Range
Source: Insects. 2021 Oct 3;12(10):904. doi: 10.3390/insects12100904 (PMC8537158; doi:10.3390/insects12100904)
Supplement: Supplementary file 1 [file insects-12-00904-s001.zip › insects-1363960-supplementary.pdf]

Supplemental Materials

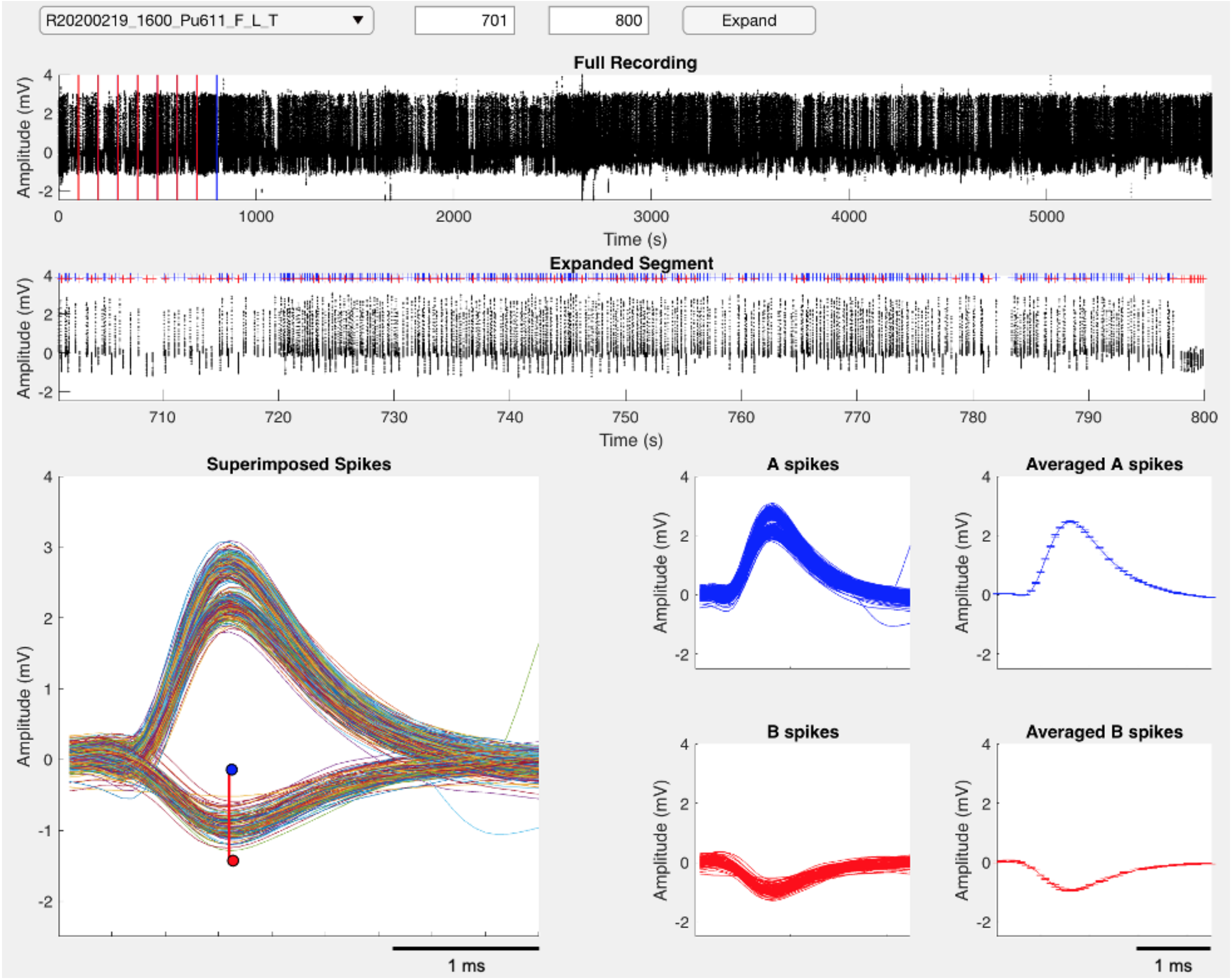

Supplemental Figure S1. Diagram of spike tagging process in MATLAB. The uppermost window shows the full, unaltered record containing all the spikes and any additional noise. The red lines indicate the 100 s segments of sequential analysis. The image shows that segment 701 to 800 s is currently selected and expanded in the second window labeled “Expanded Segment.” The window labeled “Superimposed Spikes” shows all the spikes within this 100 s interval expanded as waveforms and layered on top of each other in a 3 ms time frame. Note the multicolored A and B spike shapes. The red line connecting the two dots is user-controlled and here tags all the B waveforms, which have been isolated and colored red in the “B spikes” window. All the remaining untagged spikes have been colored blue and displayed in the “A spikes” window. The “Averaged A spikes” and “Averaged B spikes” windows show the average A and B spike waveforms, respectively, along with error bars ($\pm$ SD).

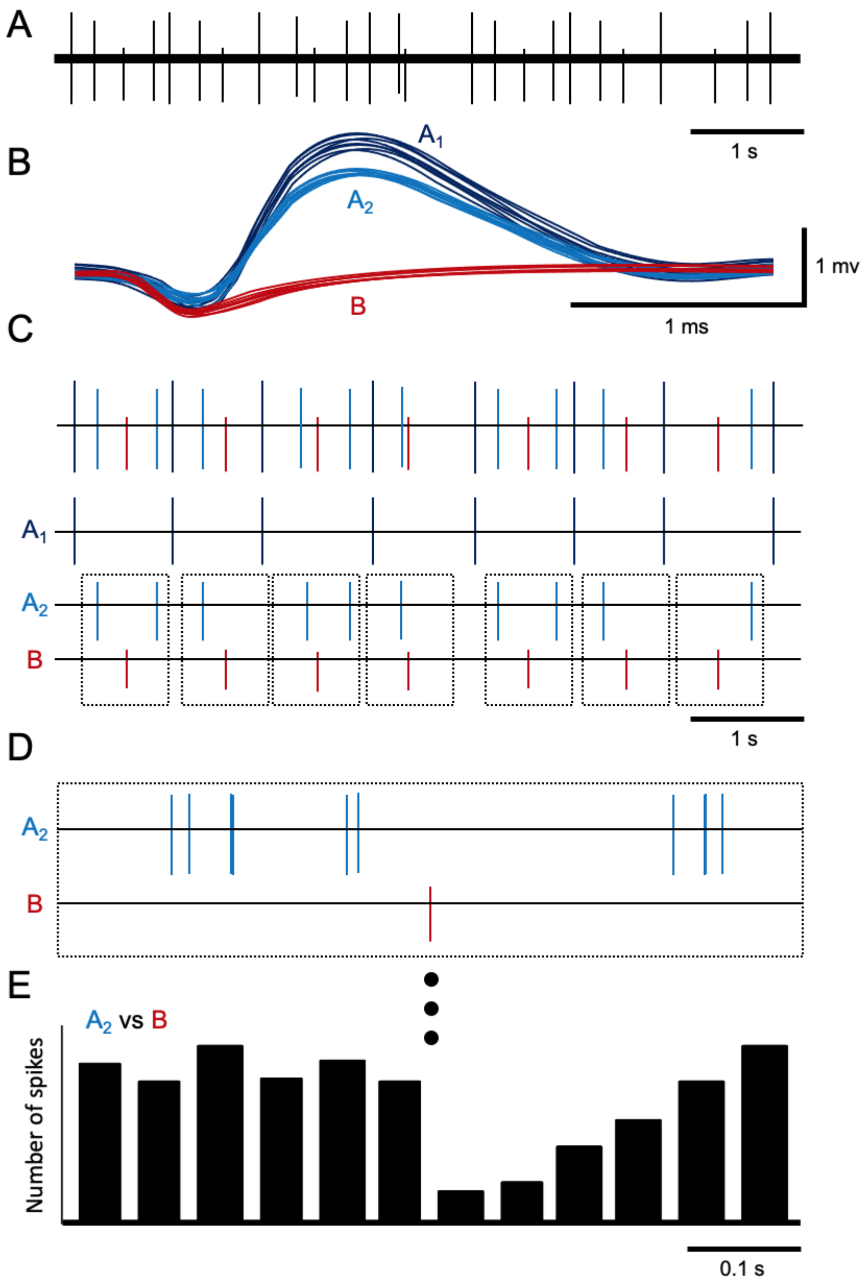

Supplemental Figure S2. Diagram of cross-correlation analysis. (**A**) A segment of an unaltered record containing all the unmarked spikes is shown in black. (**B**) The spikes identified in A have been expanded and layered on top of each other within a 3 ms time frame. Three identified spikes A_1, A_2, and B are shown in dark blue, light blue, and red respectively. (**C**) Based on the classifications, the record is now shown without the noise and the spikes color-coded. Below, the A_1, A_2, and B spikes have been separated onto individual traces. Small time windows (dashed boxes) are drawn around each B spike and all its corresponding A_2 spikes. (**D**) All the time windows from C are superimposed to show the relative firings of A_2 spikes to B spikes in the segment. (**E**) Summing all the time windows for the entire record generates a cross-correlation histogram (correlogram) of A_2 spikes relative to the B spikes. In the example shown, A_2 spike activity is suppressed following the B spikes, suggesting an inhibitory effect of B spikes on the A_2 spikes.

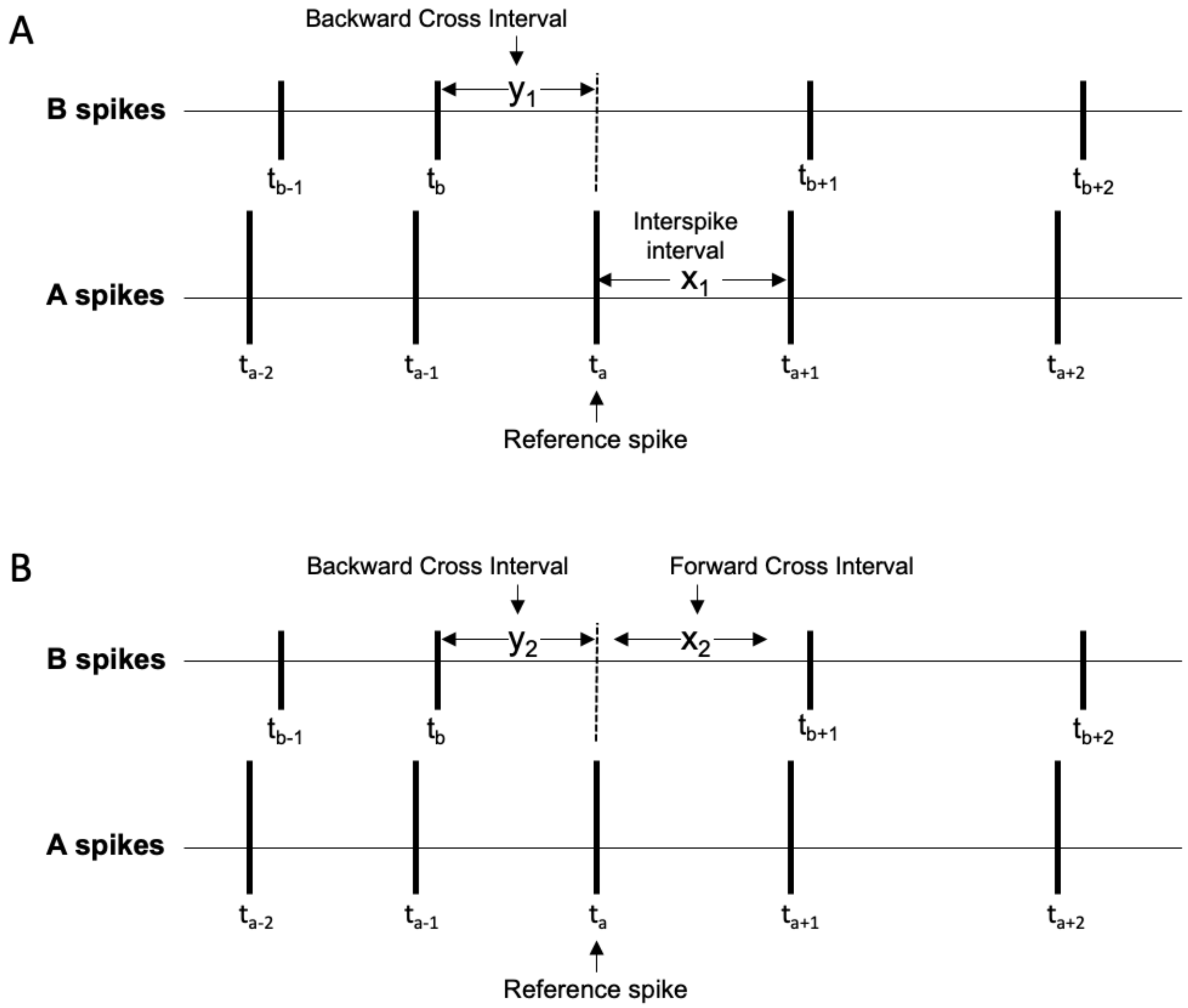

Supplemental Figure S3. Creation of conditional interspike interval and conditional cross-interval plots. (**A**) The first graphs are called conditional interspike interval plots (abbreviated CII). To make these plots, one spike train becomes the reference train and the other the cross train. First, each spike in the reference train is located and the time to the first previous spike in the cross train -- called the backward cross interval -- is noted. Next, the time to the next spike in the reference train -- which is called the interspike interval -- is noted. Finally, a scatter plot is produced by plotting the backward cross interval on the y-axis against the interspike interval on the x-axis. (**B**) The other graphs are called conditional cross-interspike plots (abbreviated CCI). In this case we are identifying the cross-train spikes both immediately preceding and succeeding the occurrence of the reference spike and plot these respectively on the y and x axis of a scatter plot. [Adapted from Tam et al. 1988]

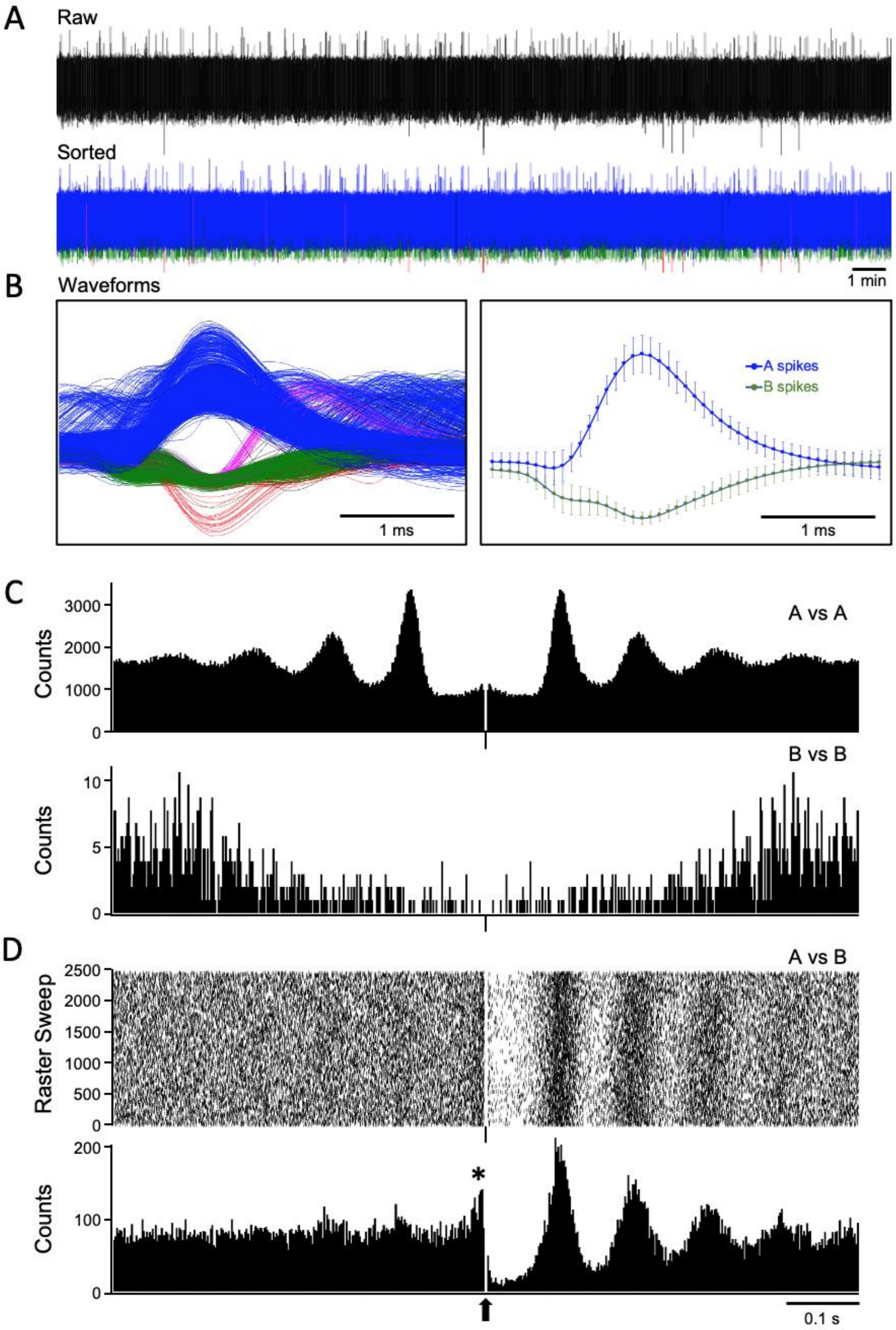

Supplemental Figure S4. Auto and cross-correlation analysis of a higher spiking frequency record. (**A**) Raw (top trace) spiking activity of a segment (~20 min) of a longer baseline recording (~45 min) a from peg sensillum of female *Paruroctonus utahensis*. The spiking frequency of the record was ~19 Hz. Spikes color coded based on waveform (bottom trace). (**B**) Superimposed waveforms from the above segment are shown in window at left; average shape of A and B spikes (± SD) are shown at right. (**C**) Auto-correlogram of A spikes (top) and B spikes (middle) from full record. (**D**) Cross-correlogram of A spikes vs B spikes from full record (bottom histogram) along with all raster sweeps (top hashmarks). Asterisk notes increased activity of A cells just prior to B cell firing (arrow).

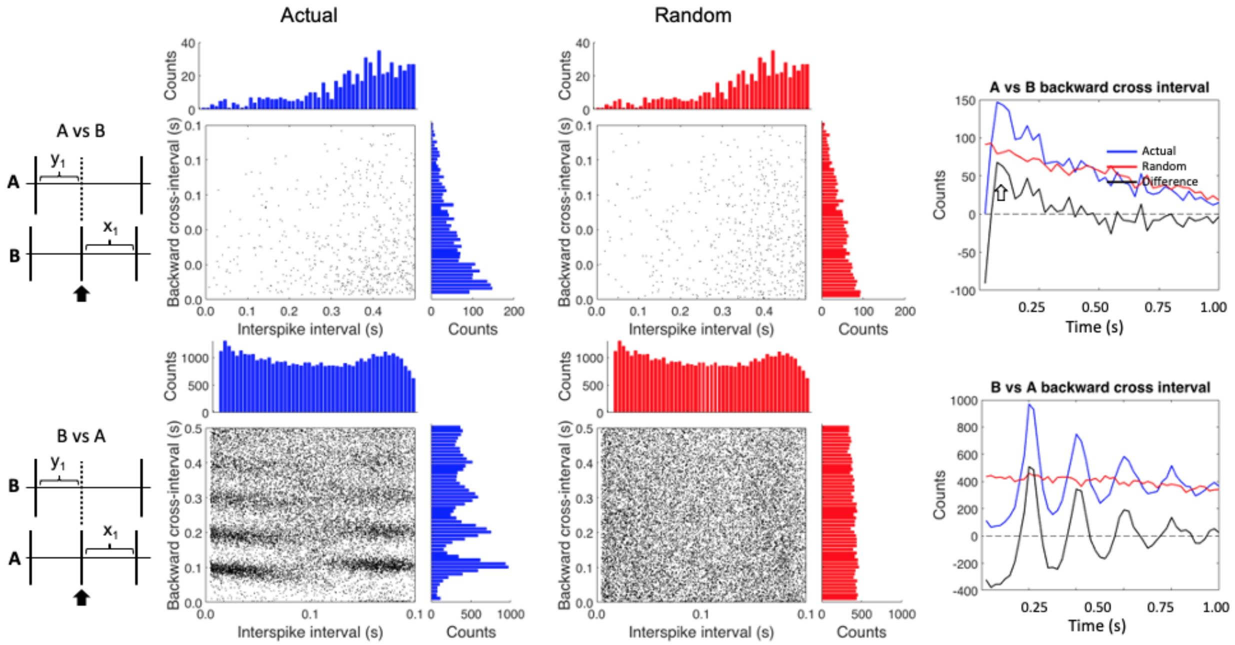

Supplemental Figure S5. Conditional interspike interval plots for higher frequency record. In the upper plots, the A spikes are referenced against the B spikes while the reverse is shown in the lower plots. The left hand (blue) plots show the actual data while the right hand (red) plots were created after randomizing the interspike intervals for each of the spike trains. The histograms on the tops and the sides of the plots show the counts by time bin for each axis. The two graphs on the right show the differences (black lines) between the actual (blue lines) and randomized data (red lines) for the backward cross intervals. The open white arrow highlights the area of increased activity in the difference plot of the A vs B backward cross-interval graph.
